# Fabrication and Evaluation of Electrospun, 3D-Bioplotted, and Combination of Electrospun/3D-Bioplotted Scaffolds for Tissue Engineering Applications

**DOI:** 10.1155/2017/6956794

**Published:** 2017-04-27

**Authors:** Liliana F. Mellor, Pedro Huebner, Shaobo Cai, Mahsa Mohiti-Asli, Michael A. Taylor, Jeffrey Spang, Rohan A. Shirwaiker, Elizabeth G. Loboa

**Affiliations:** ^1^Joint Department of Biomedical Engineering, University of North Carolina at Chapel Hill and North Carolina State University, Raleigh, NC 27695, USA; ^2^Edward P. Fitts Department of Industrial and Systems Engineering, North Carolina State University, Raleigh, NC 27695, USA; ^3^Department of Orthopaedics, University of North Carolina School of Medicine, Chapel Hill, NC 27599, USA; ^4^College of Engineering, University of Missouri, W1051 Thomas & Nell Lafferre Hall, Columbia, MO 65211, USA

## Abstract

Electrospun scaffolds provide a dense framework of nanofibers with pore sizes and fiber diameters that closely resemble the architecture of native extracellular matrix. However, it generates limited three-dimensional structures of relevant physiological thicknesses. 3D printing allows digitally controlled fabrication of three-dimensional single/multimaterial constructs with precisely ordered fiber and pore architecture in a single build. However, this approach generally lacks the ability to achieve submicron resolution features to mimic native tissue. The goal of this study was to fabricate and evaluate 3D printed, electrospun, and combination of 3D printed/electrospun scaffolds to mimic the native architecture of heterogeneous tissue. We assessed their ability to support viability and proliferation of human adipose derived stem cells (hASC). Cells had increased proliferation and high viability over 21 days on all scaffolds. We further tested implantation of stacked-electrospun scaffold versus combined electrospun/3D scaffold on a cadaveric pig knee model and found that stacked-electrospun scaffold easily delaminated during implantation while the combined scaffold was easier to implant. Our approach combining these two commonly used scaffold fabrication technologies allows for the creation of a scaffold with more close resemblance to heterogeneous tissue architecture, holding great potential for tissue engineering and regenerative medicine applications of osteochondral tissue and other heterogeneous tissues.

## 1. Introduction

Tissue engineering is a growing field that aims to create living biological substitutes to restore, repair, or regenerate native tissue or organ function that may be affected by disease or injury. The main components of engineered tissues include cells, scaffolds, and chemical and/or mechanical cues to replicate or mimic the physiological conditions of the target tissue [1]. The individual characteristics of each of these components and their interactions have a significant impact on the quality and functionality of engineered tissues [2]. As such, it is important to determine the optimum combination of relevant characteristics for any target tissue to be engineered.

The most commonly used strategies in tissue engineering involve seeding a uniform or homogenous scaffold with a single cell type. But, in reality, most tissues are composed of several cell types and a diverse and heterogenic extracellular matrix (ECM) framework [3, 4]. Failure to replicate the physiological and native conditions can have negative results in engineered tissue integration and function when implanted in an organism [1, 5]. Scaffold design in tissue engineering is particularly important, as the scaffold should not only provide an optimal 3D network to support cell adhesion and proliferation but also appropriately guide cell differentiation, when stem cells are used, to generate the desired tissue(s) [6, 7]. Osteochondral tissue engineering has proven to be very complex due to the presence of different cell types, ECM heterogeneity, and the multiple three-dimensional materials which characterize an articular joint including the following: porous subchondral bone, a transitional dense cartilage framework, and a tidemark separating the layers [4, 8]. Most scaffold fabrication techniques cannot recapitulate the heterogeneous multiphasic porous architecture that is native to an articular joint.

The goal of this study was to combine two commonly used fabrication techniques—electrospinning and 3D printing—to develop a simple and reproducible scaffold that incorporates both nano- and microscale fibrous architecture and more closely mimic heterogenous tissues. We evaluated a combined 3D printed/electrospun scaffold architecture mimicking heterogeneous tissues such as the osteochondral complex, in comparison to solely 3D printed microfibrous or solely electrospun nanofibrous scaffolds, for their ability to support viability and proliferation of human adipose-derived stem cells (hASC). Further, we also tested and compared the feasibility and efficacy of implanting full-thickness (6 mm) combined 3D printed/electrospun versus stacked electrospun scaffolds in an ex vivo porcine model using a clinically relevant procedure for osteochondral defect repair. The results show the ability to successfully engineer a scaffold that resembles the physiological thickness as well as a multiscale heterogeneous fibrous architecture of osteochondral tissue. This combined 3D printing/electrospinning approach could be extended to other tissues with heterogenous ECM framework and/or transitional tissues like ligament and tendon bone insertions in the future.

## 2. Materials and Methods

### 2.1. 3D-Bioplotting Microfibrous Scaffolds

**Figure 1:**
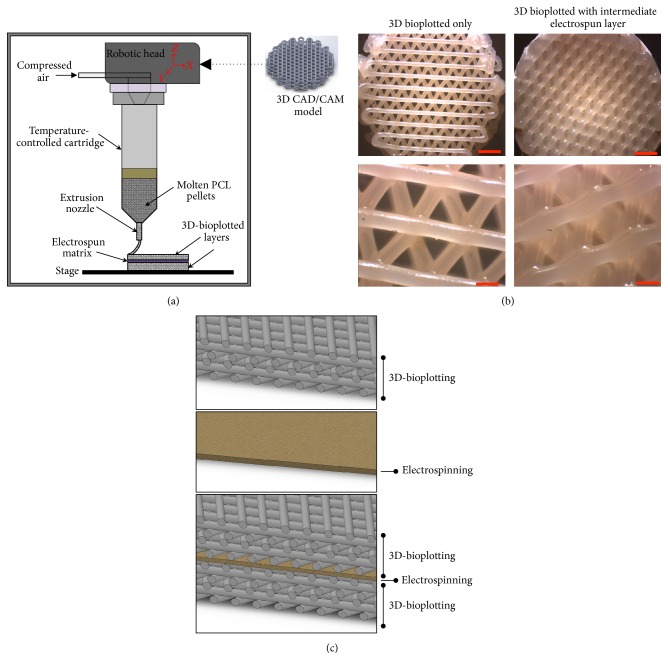
Fabrication of combined micro- and nanofibrous scaffold by sandwiching an electrospun layer between 3D-bioplotted layers. (a) Schematic of technical approach. (b) 3D-bioplotted scaffold with and without an electrospun layer (scale bars top = 2 mm; bottom = 500 *μ*m). (c) Cross-sectional CAD representation of three different scaffolds created and evaluated. Top: microfibrous scaffold fabricated using 3D bioplotting technique only. Middle: nanofibrous scaffold fabricated using electrospinning only. Bottom: alternating micro- and nanosized fibers by combining 3D bioplotting and electrospinning techniques. Colors and textures for visualization purposes only.

Thin disc-shaped scaffolds (Ø 14.5 mm × 2 mm) (Figure 1(a)) were fabricated using polycaprolactone (PCL, *M*_*W*_ = 80 K, Sigma-Aldrich Co., St. Louis, MO) on a 3D-Bioplotter (4th-Generation Developer Series, EnvisionTEC GmbH, Gladbeck, Germany). The scaffolds and their CAD model (Solidworks 2014) were designed to facilitate the fitting and culturing of finished constructs in standard 24-well cell culture plates. Previously determined optimal bioplotting process parameters were used [9–11]. In brief, PCL was extruded at an extrusion pressure of 0.5 N/mm^2^ and extrusion temperature of 180°C through a 0.4 mm inner diameter nozzle with a printing speed of 0.4 mm/s following a 45-minute preheat interval for stabilization and air removal from the melt. The scaffold design featured a separation of 1.5 mm between the axes of adjacent strands, which kept constant through all the layers, and a strand lay-down pattern of 0°/120°/240° between adjacent layers, yielding a highly interconnected pore network.

### 2.2. Electrospinning Nanofibrous Scaffolds

PCL was dissolved in chloroform and dimethylformamide (Sigma) at a ratio of 3 : 1 to create an 11% solution. The solution was mixed continuously at 80°C for at least 4 hours. The PCL solution was electrospun using an internal nozzle diameter of 0.508 mm on a static collector covered with aluminum for 3 hours immediately after preparation at a feed rate of 0.7 *μ*L/hr and spinning distance of 13–15 cm using 15 kV. The electrospun nanofibrous scaffolds were detached from the aluminum surface prior to being used.

Stacked scaffolds (6 mm thick) used for implantation were generated by stacking together multiple electrospun layers using collagen type I gel in between the layers, at a concentration of 3 mg/mL (Vitrogen, Angiotech BioMaterials Corporation, Palo Alto, CA) [12]. Collagen was first neutralized to pH 7.0, pipetted between the layers, and allowed to polymerize for 2 hours at 37°C.

### 2.3. Combined 3D-Bioplotted Microfibrous/Electrospun Nanofibrous Scaffolds

The integrated micro- and nanofibrous PCL scaffolds were fabricated using a combination of 3D-bioplotting and electrospinning. The electrospun layers were cut into 14.5 mm diameter circles to match the size of the 3D scaffolds. First, a 2 mm basal section of 3D-bioplotted scaffold was printed as mentioned above. We then placed the circular electrospun layer directly over the basal layer and continued printing another 2 mm section on top of the electrospun layer to generate a final 4 mm thick scaffold with an electrospun layer in the middle (Figure 1). These scaffolds were used for testing hASC viability and proliferation. The full thickness (6 mm) scaffolds evaluated for implantation techniques were fabricated using the same procedure but with 4 mm basal section and 2 mm top section 3D bioplotted.

### 2.4. Isolation and Expansion of Human Adipose-Derived Stem Cells

Excess adipose tissue was collected from five female premenopausal donors (ages 24 to 36) in accordance with an approved IRB protocol at UNC Chapel Hill (IRB 04-1622) [13]. Human ASC were isolated from the tissue as previously described by our lab and others [14–16]. Cells were expanded in complete growth medium (CGM) comprised of alpha-modified minimal essential medium (*α*-MEM with L-glutamine) (Invitrogen, Carlsbad CA), 10% fetal bovine serum (FBS) (Premium Select, Atlanta Biologicals, Lawrenceville GA), 200 mM L-glutamine, and 100 I.U. penicillin/100 *μ*g/mL streptomycin (Mediatech, Herndon VA). The cells were cultured at 37°C in 5% CO_2_ until reaching 80% confluency and then passaged using trypsin-EDTA (Invitrogen). A superlot was generated by pooling equal numbers of cells from the five individual donor cell lines into a single culture vessel and characterized for multilineage differentiation potential, ensuring the cells differentiated representative of an average of the five cell lines [13].

### 2.5. Seeding of Scaffolds

The 3D-bioplotted, electrospun, and combined bioplotted/electrospun disc scaffolds (Ø 14.5 mm) were designed and fabricated to fit in 24-well plates (well Ø 15.6 mm), limiting any space between the walls of the well and the periphery of the scaffold where cells could potentially migrate towards the bottom of the wells. Prior to seeding, scaffolds were sterilized for 30 minutes in 70% ethanol, rinsed three times with sterile phosphate buffered saline (PBS) and once with CGM. Due to the difference in thickness between the scaffold designs (electrospun = 200 *μ*m, 3D-bioplotted = 2 mm, and combined scaffolds = 4.2 mm,), a total of 100,000 cells were seeded in each 3D scaffold and 20,000 cells in each electrospun scaffold over a two-day period. On the first day, half of the total amount of cells (50,000 cells for 3D scaffolds and 10,000 cells for electrospun scaffolds) were resuspended in 1 mL of CGM and added to each well containing a scaffold. The cells/scaffolds were incubated overnight while gently rocking to allow cell distribution and adhesion throughout the scaffolds. On the second day, each scaffold was overturned and seeded with the remaining cells to allow adhesion of cells on both sides of the scaffolds. Once again, the scaffolds were incubated overnight while gently rocking. The scaffolds were then transferred to a new well for performance of all assays.

### 2.6. Cell Viability Analyses

Seeded scaffolds were cultured in CGM for 21 days to promote growth and proliferation. After 21 days of culture, a LIVE/DEAD viability assay (Life Technologies) was performed per the manufacturer's instructions on all scaffolds to assess hASC viability within the scaffolds. Briefly, hASC-seeded scaffolds were gently washed with sterile PBS three times, and then 500 *μ*L of 2 *μ*M calcein AM and 4 *μ*M EthD-1 solution was added to each scaffold and incubated for 30 minutes. Scaffolds were visualized using a Leica DM5500B Fluorescent Microscope and the compatible LAS-AF software. Two consecutive images per section were taken to visualize live cells (green) and dead cells (red), and a composite overlaid image was generated to visualize both channels in the same frame. To optimize exposure, gain, and intensity parameters, the LUT function of the software was used. All images were taken at 10x magnification.

### 2.7. Cell Proliferation Analyses

Cell proliferation was assessed (*n* = 4 scaffolds per time point) at days 1, 4, 7, 11, and 20 after seeding using the AlamarBlue colorimetric assay (Life Technologies). Acellular scaffolds were also analyzed as controls, and all data was normalized to the appropriate acellular control scaffold. At each time point, a 1 : 10 ratio of AlamarBlue : CGM solution was added to each scaffold and incubated at 37°C and 5% CO_2_ for 3 hours. After incubation, absorbance was measured at wavelengths of 570 nm and 600 nm using a Microplate reader (Tecan Group Ltd., Männedorf, Switzerland) and the Magellan Data Analysis Software (Tecan Group Ltd.).

### 2.8. Implantation of Scaffolds in a Porcine Model

Cadaveric porcine knees were utilized to create a suitable ex vivo environment in a large animal model that resembles the human knee. This model has been used extensively in vivo to evaluate articular cartilage repair techniques [17, 18]. Using current human surgical techniques and currently utilized hardware (COR Osteochondral Autograft Transfer System, DePuy Mitek, Raynham, MA), stacked electrospun scaffolds and a single 3D-bioplotted scaffold were implanted into osteochondral defects created via drilling to evaluate scaffold-handling characteristics in the surgical setting. Osteochondral Autograft Transfer techniques commonly employed for human patients were utilized [19–21]. Briefly, a power reamer was used to create an osteochondral defect to a depth of 8 mm with an 8 mm diameter. Using the donor cutting tool from the COR system, an 8 mm diameter section of the osteochondral stacked scaffold was cut from a 14.5 mm diameter scaffold (typical size created using our approach). The scaffold was then implanted into the recipient hole per the recommended COR Osteochondral Autograft Transfer System technique (DePuy Mitek, Raynham, MA), consistent with current human surgical procedures. Optimal scaffold depth was selected based on the handling ability of the scaffold and successful implantation of the scaffolds to fill the created defect.

### 2.9. Statistical Analysis

Statistical analysis was performed using Prism (version 6.07, GraphPad Software). Bar graphs are represented as mean ± SEM. Differences were determined using a one-way ANOVA with Tukey post hoc test. A level of *p* < 0.05 was considered significant.

## 3. Results

### 3.1. Cell Viability, Migration, and Proliferation in Scaffolds

**Figure 2:**
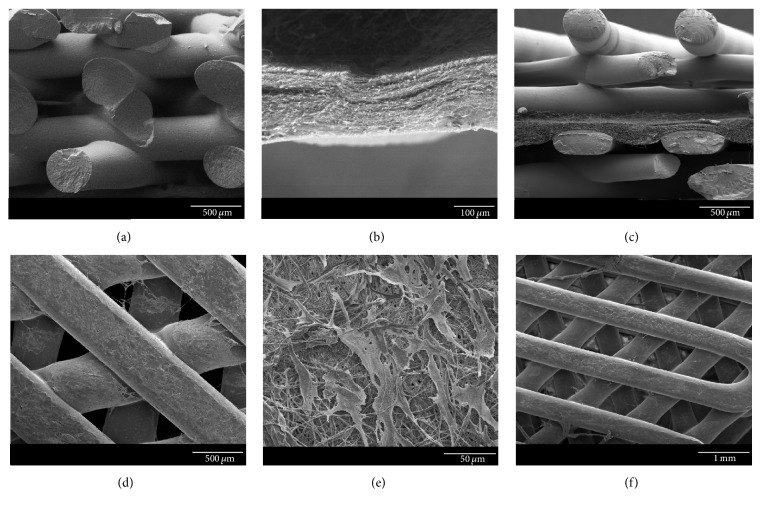
Scanning electron microscopy of (a) 3D-bioplotted scaffold; (b) electrospun nanofibers; (c) combined 3D-bioplotted and electrospun scaffolds (electrospun layer in middle); (d) cells growing on 3D-bioplotted scaffold; (e) cells growing on electrospun nanofibers; and (f) cells growing on combined 3D and electrospun scaffold (scale bars (a), (c), (d) = 500 *μ*m; (b) = 100 *μ*m; (e) = 50 *μ*m; (f) = 1 mm).

Scanning electron microscopy (SEM) images show the different fiber size and arrangement between 3D-bioplotted and electrospun scaffolds (Figures 2(a) and 2(b)), as well as the combined nano/microfibrous scaffold (Figure 2(c)). Cells can also be observed on the surface of all three seeded scaffolds (Figures 2(d)–2(f)).

**Figure 3:**
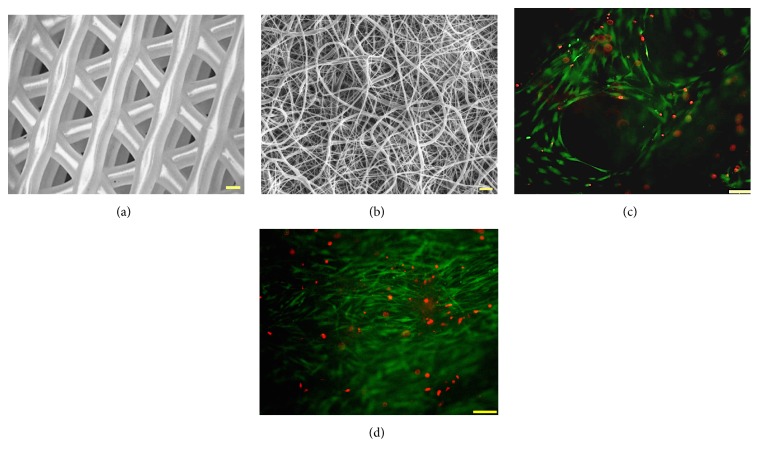
Cell viability and proliferation of human adipose-derived stem cells seeded on (a, c) 3D-bioplotted scaffolds and (b, d) electrospun scaffolds (green = live cells; red = dead cells).

To ensure cells could grow and proliferate throughout individual nano- and microfibrous scaffolds, we first measured and compared hASC proliferation and viability after 21 days in culture in both electrospun and 3D-bioplotted scaffolds. Cells were able to adhere and proliferate in both micro- and nanofibrous scaffolds, with minimal dead cells observed after 21 days in culture. However, the hASC exhibited higher proliferation and more uniform spreading in electrospun scaffolds when compared to 3D-bioplotted scaffolds (Figure 3).

**Figure 4:**
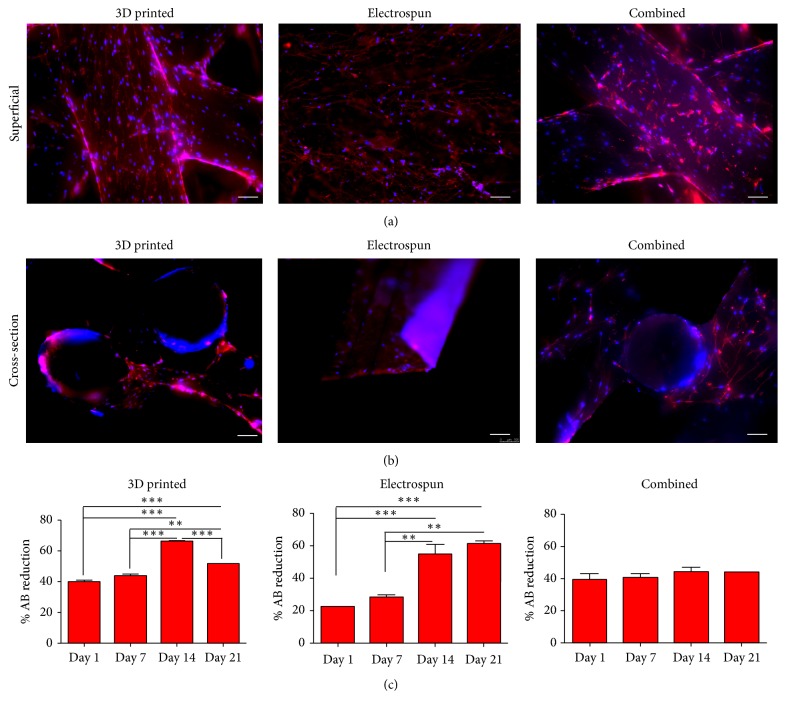
Cell spreading and proliferation of human adipose-derived stem cells throughout scaffolds. Cells were cultured for 21 days in 3D-bioplotted, electrospun, or combination of 3D-bioplotted/electrospun scaffolds, fixed, and stained (actin = red; nuclei = blue). Superficial and cross-sectional views show cells present both on the surfaces of the scaffolds (superficial) and throughout the centers of the scaffolds on the 3D-bioplotted and combined scaffolds (cross-section). Human ASC exhibited steady proliferation over 21 days of culture on all scaffold types as indicated by AlamarBlue (% AB reduction). Bars indicate mean ± SEM (^*∗∗∗*^*p* < 0.0001; ^*∗∗*^*p* < 0.005).

We then compared cell proliferation and migration in all three scaffolds (Figure 4). Cells were visible on the superficial layers of all three scaffolds (Figure 4(a)) and throughout the scaffolds (Figure 4(b)). Cells seeded on 3D scaffolds only had significantly increased proliferation after 14 days in culture, with a decrease at day 21. Electrospun scaffolds had significant increase in proliferation after 14 days in culture, and the combined scaffold had a steady proliferation without a decline over the 21-day culture period (Figure 4(c)).

### 3.2. Comparison of Implantation of Micro- and Nanofibrous Scaffolds in a Porcine Ex Vivo Model

**Figure 5:**
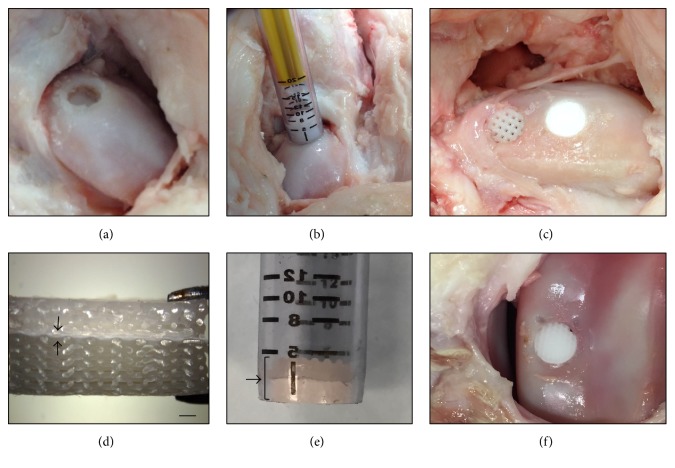
Implantation technique of 3D-bioplotted, electrospun, and combined 3D-bioplotted/electrospun scaffolds into a cadaveric porcine knee. (a) A power reamer was used to create an osteochondral defect to a depth of 8 mm and 8 mm diameter. (b) The COR system was used to implant the scaffolds into the osteochondral defect. (c) View of 3D-bioplotted scaffold (left) and electrospun scaffold (right) after implantation. (d) Combined 3D-bioplotted/electrospun scaffold prior to implantation (black arrows indicate electrospun layer, scale bar = 1 mm). (e) Combined 3D-bioplotted/electrospun scaffold inserted into the COR system for implantation (bracket and arrow pointing at scaffold inside the device). (f) Combined 3D-bioplotted/electrospun scaffold after implantation.

Standard human operative techniques were used to implant both stacked nanofibrous scaffolds and combination of 3D-bioplotted/electrospun scaffolds into a cadaveric porcine knee model, to determine the translational applicability of these scaffolds in a relevant in vivo model for osteochondral tissue engineering. Due to the limited thickness of each electrospun layer (approximately 200 *μ*m), 30 different electrospun layers (each Ø 14.5 mm) were stacked using collagen I in between each layer as previously described [22], to create a 6 mm thick scaffold. A 3D-bioplotted/electrospun scaffold (6 mm thick; 14.5 mm diameter) was also fabricated for implantation into an osteochondral defect and to compare to the implantation technique of the electrospun stacked scaffold. The COR Osteochondral Autograft Transfer System technique was compatible for sizing and implanting both stacked electrospun and 3D-bioplotted scaffolds. However, we found that stacked electrospun scaffolds easily delaminated when using the plug harvest system and needed to be frozen prior to implantation to prevent delamination. The 3D-bioplotted scaffolds and the combined 3D-bioplotted/electrospun scaffolds were easily inserted using the COR system and successfully implanted into the cadaveric porcine knee without delamination (Figure 5).

## 4. Discussion

Electrospinning is a commonly used technique in tissue engineering allowing for production of a dense framework of fibers with pore sizes and fiber diameters that closely resemble the architecture of native ECM [23–25]. However, this technique has limitations in generating three-dimensional structures of relevant physiological thicknesses. 3D-printing processes such as 3D-bioplotting have emerged in the last decade as alternative techniques to generate scaffolds of physiologically relevant thicknesses and morphologies that are biomimetic to different tissues and organs [5, 26]. Although these techniques have better three-dimensional geometrical flexibility, they are limited to generation of microsized fibers and larger pore sizes than those of electrospun scaffolds. Our goal here was to develop a methodology to create a multiscale scaffold design using a combination of electrospinning and 3D-bioplotting in order to better match the architecture of heterogenous tissue, using osteochondral tissue as a sample model.

Previous investigators have also attempted to combine micro- and nanofibrous architecture into a single scaffold for different tissue engineering applications. For example, Yeo and Kim created cell-laden hierarchical scaffolds that incorporated microsized fibers for support, combined with electrospun nanofibers to enhance cell proliferation and distribution [27]. Their approach incorporated cell-laden alginate struts of osteoblast-like cells (MG63) to obtain homogenous cell distribution within the scaffold. Although they successfully achieved homogenous cell proliferation throughout the scaffold, their complex fabrication procedure required repetition of three different techniques including melt-dispensing, followed by electrospinning, and cell-dispensing in order to obtain cell-laden alginate struts. Their goal was to create a homogenous tissue with even cell distribution that could be used as a promising scaffold for regeneration of soft and hard tissue, but not necessarily a heterogenous tissue.

In this study, we present a facile and reproducible technique to develop an integrated approach combining both electrospun nanofibers and 3D-plotted microfibers to recapitulate the heterogenous architecture of native tissue. In the first approach, we electrospun nanofibers over a 3D-bioplotted scaffold. Although we successfully coated the 3D-bioplotted scaffold with electrospun nanofibers, the two layers delaminated during culture of the combined micro/nanofibrous scaffold in cell culture medium (data not shown). Such delamination would likely be a greater problem in vivo; therefore we believe this technique is not appropriate for generating a combined nano/microfibrous scaffold.

We therefore evaluated an alternative approach by 3D-bioplotting directly on an electrospun scaffold. An electrospun nanofibrous layer was placed directly over a freshly printed 3D scaffold, and then another 3D scaffold was printed on top of this electrospun layer (Figure 1(a)). This combined micro/nanofibrous scaffold did not delaminate during culture. It is possible that the heat from the printed microfibers facilitated binding of the nanofibers to the printed microfibers; however, as observed in the SEM images of the combined scaffold (Figure 2(c)), the heat did not seem to impact the fiber morphology. Increased thickness can be achieved by continued layering of the materials, alternating between electrospun nanofibers and 3D-bioplotted microfibers, to create a multilayered micro/nanofibrous scaffold as needed. Based on our findings, the best approach to fabricate a combined micro/nanofibrous scaffold was to 3D print over electrospun layers. This combined technique produced single scaffolds that more closely resembled the native heterogenous architecture.

Cell proliferation was significantly increased over time in both 3D-bioplotted and electrospun scaffolds. Although proliferation did not significantly increase over time in the combined scaffolds, we still observed a steady proliferation with no decline over time. Although cell proliferation and viability were higher in the electrospun scaffolds, this technique has thickness and 3-dimensional limitations, typically resulting in creation of a ~200-micron scaffold after hours of electrospinning using conventional electrospinning systems. In addition, stacking several electrospun layers to create a thicker scaffold not only is time consuming but can also limit the migration of cells throughout the layers (data not shown). We first tested hASC cell viability and migration throughout micro- and nanofibers by seeding cells on scaffolds with either the electrospun layer on top or underneath the 3D-bioplotted layer. We observed that when cells were seeded on the 3D-bioplotted scaffold, the cells proliferated and migrated down to the electrospun layer. However, if the cells were seeded over the electrospun layer, the nanofiber acted as a barrier and prevented cells from migrating down into the 3D-bioplotted layer.

With our technique, the electrospun membrane can be used to separate layers that require different cell types in heterogenous tissues, like bone and cartilage in osteochondral tissue. This way, the chondrocytes, for instance, will not migrate into the underlying subchondral bone layer or vice versa, while providing a natural framework that resembles the tight collagen network in that area.

Ex vivo handling of 3D-bioplotted, stacked electrospun, and combined 3D-bioplotted/electrospun constructs confirmed that they morphologically approximated the current human tissue utilized for autologous osteochondral transfer within human joints. Using clinically relevant surgical techniques and commercially available hardware, acellular scaffolds comprised of all three designs were successfully implanted in situ using a porcine cadaveric knee. However, the stacked electrospun nanofibrous scaffolds generated by collagen binding of multiple single electrospun layers easily delaminated when implanted in the porcine knee. These stacked scaffolds needed to be frozen prior to implantation in order to prevent delamination. This could be a critical factor when dealing with seeded scaffolds and can affect cell viability. The 3D-bioplotted and combined 3D-bioplotted/electrospun scaffolds allowed for creation of clinically relevant thicknesses (5–7 mm) and were easily implanted using standard surgical procedures without delamination or breakage. This suggests a facile implementation of current autograft human osteochondral techniques to implant such multiphasic osteochondral scaffolds, indicating the immediate potential clinical translatability of our proposed combined micro- and nanofibrous scaffolds.

## 5. Conclusion

Creation and utilization of appropriate scaffold architecture are a critical step towards generation of an engineered tissue construct that mimics complex native tissue. Electrospun nanofibrous scaffolds, with their dense framework, pore sizes, and fiber diameters, have limitations for creation of three-dimensional structures of relevant physiological thicknesses. We compared these nanofibrous scaffolds to 3D-bioplotted scaffolds, constructs with better dimensional control and reproducibility but thicker fibers and larger pore sizes. We combined these two fabrication approaches, with results indicating that a combination of 3D-bioplotted and electrospun scaffolds could provide an excellent alternative for full heterogenous tissue regeneration. We tested our scaffolds in a relevant implantation model for osteochondral tissue and showed that although electrospun scaffolds yield higher cell proliferation, they are hard to manipulate during a clinically relevant osteochondral transplantation technique. Our combined scaffold was easily implanted using common surgical procedures into an osteochondral defect, without delamination or breaking of the scaffold.

This is one of the first studies to combine two commonly used scaffold fabrication technologies into a simple scaffold to more closely match thicker tissues with heterogenous matrix architecture. Such approaches may hold great potential for tissue engineering and regenerative medicine applications.
